# Ferroptosis-related lncRNA signature predicts prognosis and immunotherapy efficacy in cutaneous melanoma

**DOI:** 10.3389/fsurg.2022.860806

**Published:** 2022-07-21

**Authors:** Yujian Xu, Youbai Chen, Zehao Niu, Zheng Yang, Jiahua Xing, Xiangye Yin, Lingli Guo, Qixu Zhang, Yi Yang, Yan Han

**Affiliations:** ^1^ Department of Plastic and Reconstructive Surgery, Chinese PLA General Hospital, Beijing, China; ^2^ Department of Plastic Surgery, The University of Texas MD Anderson Cancer Center, Houston, TX, United States; ^3^ Department of Dermatology, Chinese PLA General Hospital, Beijing, China

**Keywords:** cutaneous melanoma, ferroptosis, immune infiltration, immune checkpoint, long non-coding RNA

## Abstract

**Purpose:**

Ferroptosis-related lncRNAs are promising biomarkers for predicting the prognosis of many cancers. However, a ferroptosis-related signature to predict the prognosis of cutaneous melanoma (CM) has not been identified. The purpose of this study was to construct a ferroptosis-related lncRNA signature to predict prognosis and immunotherapy efficacy in CM.

**Methods:**

Ferroptosis-related differentially expressed genes (FDEGs) and lncRNAs (FDELs) were identified using TCGA, GTEx, and FerrDb datasets. We performed Cox and LASSO regressions to identify key FDELs, and constructed a risk score to stratify patients into high- and low-risk groups. The lncRNA signature was evaluated using the areas under the receiver operating characteristic curves (AUCs) and Kaplan-Meier analyses in the training, testing, and entire cohorts. Multivariate Cox regression analyses including the lncRNA signature and common clinicopathological characteristics were performed to identify independent predictors of overall survival (OS). A nomogram was developed for clinical use. We performed gene set enrichment analyses (GSEA) to identify significantly enriched pathways. Differences in the tumor microenvironment (TME) between the 2 groups were assessed using 7 algorithms. To predict the efficacy of immune checkpoint inhibitors (ICI), we analyzed the association between *PD1* and *CTLA4* expression and the risk score. Finally, differences in Tumor Mutational Burden (TMB) and molecular drugs Sensitivity between the 2 groups were performed.

**Results:**

We identified 5 lncRNAs (AATBC, AC145423.2, LINC01871, AC125807.2, and AC245041.1) to construct the risk score. The AUC of the lncRNA signature was 0.743 in the training cohort and was validated in the testing and entire cohorts. Kaplan-Meier analyses revealed that the high-risk group had poorer prognosis. Multivariate Cox regression showed that the lncRNA signature was an independent predictor of OS with higher accuracy than traditional clinicopathological features. The 1-, 3-, and 5-year survival probabilities for CM patients were 92.7%, 57.2%, and 40.2% with an AUC of 0.804, indicating a good accuracy and reliability of the nomogram. GSEA showed that the high-risk group had lower ferroptosis and immune response. TME analyses confirmed that the high-risk group had lower immune cell infiltration (e.g., CD8+ T cells, CD4+ memory-activated T cells, and M1 macrophages) and lower immune functions (e.g., immune checkpoint activation). Low-risk patients whose disease expressed *PD1* or *CTLA4* were likely to respond better to ICIs. The analysis demonstrated that the TMB had significantly difference between low- and high- risk groups. Chemotherapy drugs, such as sorafenib, Imatinib, ABT.888 (Veliparib), Docetaxel, and Paclitaxel showed Significant differences in the estimated IC50 between the two risk groups.

**Conclusion:**

Our novel ferroptosis-related lncRNA signature was able to accurately predict the prognosis and ICI outcomes of CM patients. These ferroptosis-related lncRNAs might be potential biomarkers and therapeutic targets for CM.

## Introduction

Cutaneous melanoma (CM), which is characterized by the malignant transformation and over-proliferation of melanocytes, has an increasing incidence and accounts for more than 75% of skin cancer-related deaths (1, 2). Although most cases of primary localized CM can be cured with surgical resection, advanced metastatic CM has a poor prognosis. The 5-year survival rates of patients with localized, regionally metastatic, and distantly metastatic CM are 98%, 63%, and 16%, respectively (3). Current prognosis prediction in CM patients depends primarily on clinicopathological factors (e.g., patient demographics, Breslow thickness, ulceration, TNM stage). Although these factors are useful for treatment decisions, they are not appropriate for the early identification of high-risk patients owing to molecular and tumor microenvironment (TME) heterogeneity (4, 5). Recent studies have shown that targeted therapy (e.g., BRAF inhibitors, MEK inhibitors) and immunotherapy with immune checkpoint inhibitors (ICIs) targeting cytotoxic T-lymphocyte antigen-4 (CTLA4) and programmed cell death protein 1 (PD1) can significantly improve the survival of CM patients (6). However, many CM patients have primary or acquired resistance to ICIs. Therefore, it is critical to identify novel molecular biomarkers to better predict the prognosis of CM patients and improve the efficacy of immunotherapy in this population.

Ferroptosis, a type of programmed cell death characterized by the accumulation of reactive oxygen species caused by iron accumulation and lipid peroxidation (7), have been proved to be different from apoptosis, necroptosis and pyroptosis (8). Ferroptosis affects cell proliferation, metastasis, and immune response in cancers. Because ferroptosis-related genes have important regulatory roles in cancer progression, they have been used to construct risk scores to predict the prognosis of many cancers, including melanoma based on mRNA expressions (9, 10). However, existing ferroptosis-related gene signatures have a poor predictive ability for the prognosis of CM patients in the most recent studies. Therefore, signatures based on ferroptosis-related non-coding RNAs may be better prognostic biomarkers owing to their higher cell/tissue specificity than protein-coding genes.

Long non-coding RNAs (lncRNAs) are a group of endogenous non-protein-coding RNAs that regulate many cellular processes, including proliferation, differentiation, migration, and invasion. They are also involved in innate and adaptive immunity by mediating the TME, and regulate ferroptosis in cancers. In addition, ferroptosis-related lncRNA signatures are useful predictors of the prognosis of many cancers, including lung adenocarcinoma (11–13), breast cancer (14, 15), gastric cancer (16, 17), bladder cancer (18), colon cancer (19), hepatocellular carcinoma (19), head and neck squamous cell carcinoma (20), and glioma (21). Ferroptosis-related lncRNAs may also have value in predicting CM prognosis. However, a ferroptosis-related lncRNA signature to predict the prognosis of CM has not yet been identified.

In the present study, we aimed to (1) identify a ferroptosis-related lncRNA signature to differentiate high- and low-risk CM patients; (2) construct a nomogram including the lncRNA signature to predict the survival of CM patients; and (3) identify differences in the TME between high- and low-risk patients and predict their responses to ICIs.

## Materials and methods

### Data acquisition

**Scheme 1:**
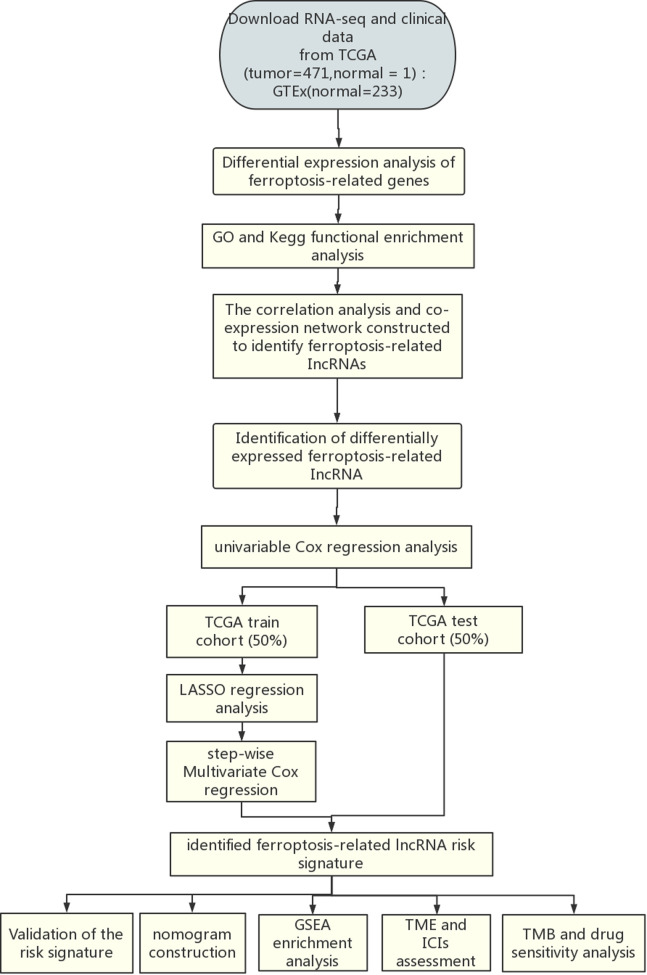
Flowchart of the analysis process.

The flow chart of the present study is shown in Scheme 1. The fragments per kilobase of per million (FPKM) of CM transcriptome, lncRNA counts data and corresponding clinical data of CM were downloaded from TCGA database (https://tcga-data.ncinih.gov/tcga/). The transcriptome samples included 471 tumor samples and 1 normal sample. Among them, 461 clinical samples were included for prognosis analysis because 10 patients had missing survival time. The clinical characteristics of the 461 patients are displayed in Supplementary Table S1. In addition, 233 normal skin samples with transcriptome data were downloaded from GTEx database (https://gtexportal.org/home/), then normalized and processed with TCGA biolinks package (22). The batch effects between TCGA and GTEx data were eliminated by R software package “limma”. The final datasets contained RNA expression profile of 471 CM samples and 234 normal samples. Sixty ferroptosis-related genes (Supplementary Table S2) that have been validated in previous studies (23–25) were obtained from the FerrDb database (26).

### Identification of ferroptosis-related differentially expressed genes and lncRNAs

To identify the ferroptosis-related differentially expressed genes (FDEGs) between tumor and normal samples, we analyzed the datasets using the classical Bayesian algorithm in the “limma” package in R (27). The ggplot2 package was used to create heatmap plots (28). We performed Gene Ontology (GO) and Kyoto Encyclopedia of Genes and Genomes (KEGG) enrichment analyses to evaluate the biological processes and pathways associated with these FDEGs. Analyses of Pearson correlation between lncRNAs and FDEGs were performed to identify ferroptosis-related lncRNAs. The association was considered significant if the square of the correlation coefficient |R^2^| > 0.4 and *P* < 0.001. Cytoscape software 3.8.0 was used to visualize the network of gene-lncRNA co-expression (29). All ferroptosis-related lncRNAs in the co-expression network were included in the differential expression analysis. Ferroptosis-related differentially expressed lncRNAs (FDELs) (false discovery rate [FDR] < 0.05 and log2FC ≥ 1) were identified using the “limma” package and illustrated in heatmap and volcano plots

### Construction of the ferroptosis-related lncRNA signature

We first performed univariate Cox regression to identify candidate FDELs that were significantly associated with overall survival (OS). We then performed least absolute shrinkage and selection operator (LASSO) regression to identify the key FDELs based on the optimal lambda value. We performed stepwise multivariate Cox regression including these key FDELs to construct a risk score. The risk score was calculated as follows: risk score $=\sum_{i=1}^{n}\beta_{i}*$ (expression of lncRNA_i_), where n is the number of key lncRNAs and *β* is the regression coefficient. Using the median risk score as a cutoff value, we classified all patients as high-risk (≥median risk score) or low-risk (<median risk score).

### Validation of the ferroptosis-related lncRNA signature

All 461 CM samples were randomly divided into a training cohort (*n* = 231) and a test cohort (*n* = 230) using the caret package in R. The detailed baseline characteristics of the training and testing cohort showed no statistical significance (Supplementary Table S3). The receiver operating characteristic (ROC) curves were applied and area under the curves (AUCs) were calculated to evaluate the accuracy of the prognostic lncRNA signature in the training, test, and entire cohorts. The risk level distribution and survival status distribution were illustrated. Kaplan-Meier analyses and log-rank tests were performed to compare survival differences between the high- and low-risk groups. In addition, principal component analysis (PCA) (30) and t-distributed stochastic neighbor embedding (t-SNE) (31) were used for dimensionality reduction and clustering visualization based on the risk score.

### Independence predictor identification and nomogram construction

To determine whether the risk score was an independent predictor of survival, we performed univariate and multivariate Cox regression analyses, which included the risk score, patient age, patient sex, melanoma stage, and TNM stage (T: tumor size; N: lymph node involvement; M: metastasis), in the entire cohort. Hazard ratios (HRs) and 95% confidence intervals (CIs) for each predictor were calculated. AUCs for 3-year OS predicted with the independent risk factors as shown by multivariate Cox regression were calculated to evaluate the predictive value of the lncRNA signature. We also developed a nomogram that included the abovementioned risk factors for the practical prediction of 1-, 3-, and 5-year OS. The performance of the nomogram was evaluated using a calibration curve analysis and C-index.

### Gene set enrichment analysis

We performed gene set enrichment analysis (GSEA) (32) to identify significantly differentially enriched pathways between the high- and low-risk groups. Gene expression was used as a phenotype label (33). The number of random sample permutations was set at 1,000. Statistical significance was set at a |normalized enrichment score| >1, nominal *P*-value <0.05, and FDR q-value <0.25 (34).

### TME assessment

The tumor purity, ESTIMATE score, immune score, and stromal score for each melanoma sample were calculated using the ESTIMATE algorithm (17). In addition, the single-sample GSEA (ssGSEA) (35), TIMER, CIBERSORT (36), MCPcounter, QUANTISEQ, XCELL, and EPIC algorithms were used to compare cellular components and immune responses between high- and low-risk groups. The differences in immune cell infiltration were shown in heatmap, violin, and radar plots. Furthermore, the ssGSEA scores of the 16 most commonly involved immune cells and 13 most commonly involved immune functions in TME were compared between the 2 groups.

### ICI efficacy

To predict the efficacy of ICIs, we systematically searched the ICI-related gene expression profiles and identified 44 ICI-related genes that might be correlated with ICI response. Among these genes, *PD1* and *CTLA4* were previously reported to be crucial targets of ICIs in CM (6, 37). The association between the expression of the *PD1* and *CTLA4* genes and the risk score was analyzed using data from The Cancer Immunome Atlas data (https://tcia.at/) to investigate the role of ferroptosis in the efficacy of ICIs.

### Tumor Mutational Burden (TMB) and drugs sensitivity analysis

The somatic mutational profile of CM was downloaded from the TCGA. The quantity and quality of the gene mutations were analyzed in the 2 groups with the “Maftools” package in R (38). In addition, we used the “pRRophetic” package in R to evaluate the half maximal inhibitory concentration (IC50) of common chemotherapy and molecular drugs.

### Statistical analysis

All statistical analyses were performed using R version 3.6.1 (Institute for Statistics and Mathematics, Vienna, Austria; https://www.r-project.org) and its (packages: limma, impute, bioconductor, ggplot2, rms, glmnet, preprocess Core, forest plot, survminer, survival ROC, beeswarm, etc.) as described previously (39). Normally and non-normally distributed variables were analyzed using the unpaired Student' t-test and the Wilcoxon test, respectively. The Benjamin-i-Hochberg method was used to identify FDELs based on FDR. All statistical tests were 2-sided with *P* ≤ 0.05 being statistically significant.

## Results

### Identification of FDEGs and FDELs

**Figure 1:**
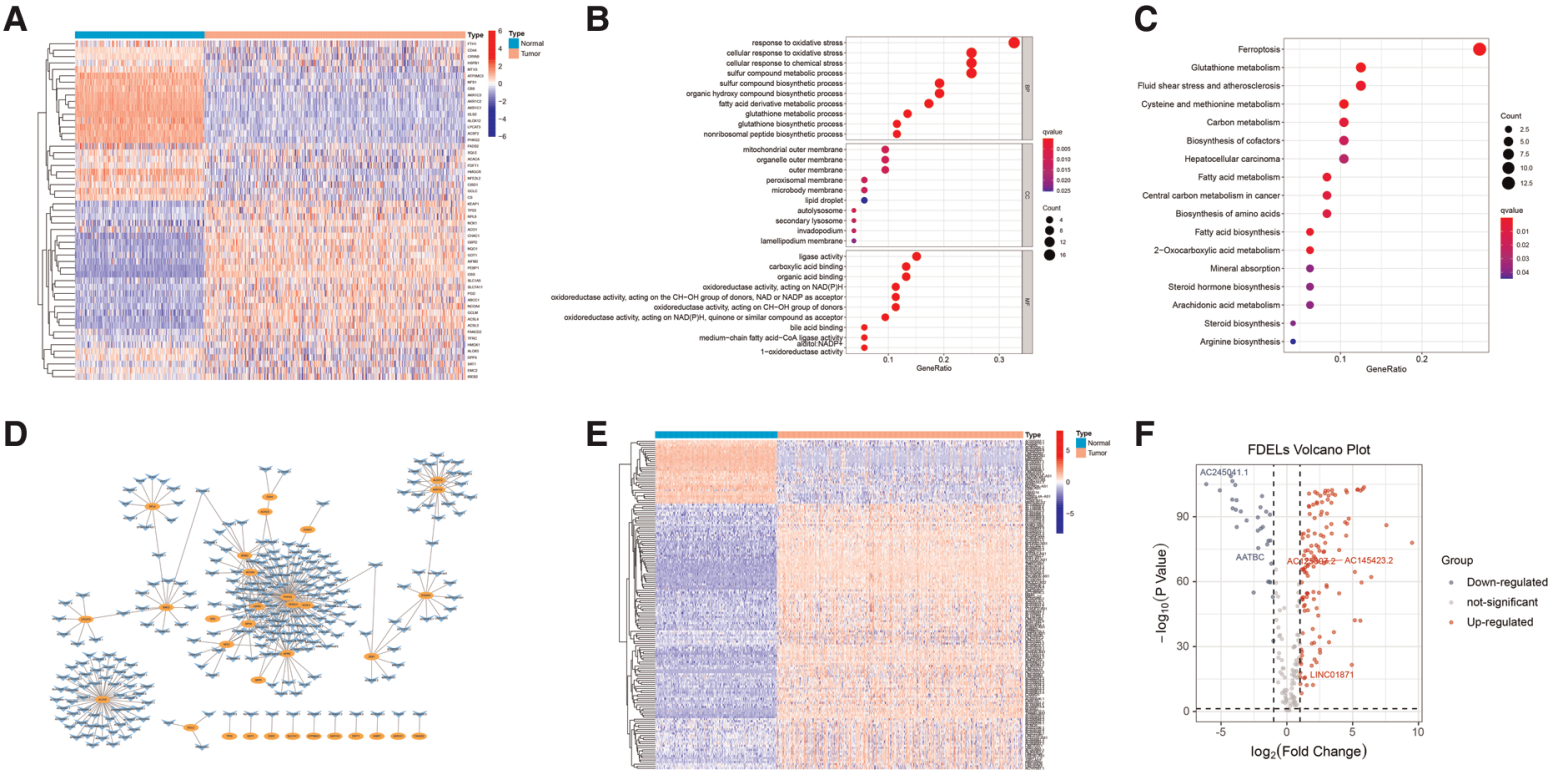
FDEG and FDEL identification and functional enrichment analyses. (**A**) The heatmap illustrates 53 FDEGs. (**B**) GO analysis of the FDEGs. (**C**) KEGG analysis of the FDEGs. (**D**) Co-expression network of FDEGs and FDELs. Yellow nodes indicate FDEGs; blue arrows FDELs. (**E,F**) A heatmap and volcano plot show 161 FDELs and 5 studied LncRNAs.

We identified 53 FDEGs (25 upregulated and 28 downregulated) between the normal and tumor samples (Figure 1A). The results of GO (Figure 1B) and KEGG (Figure 1C) analyses indicated that FDEGs were mainly enriched in ferroptosis-related biological processes (e.g., response to oxidative stress) and signaling pathways (e.g., ferroptosis). Pearson correlation analysis identified 387 ferroptosis-related lncRNAs (326 upregulated and 61 downregulated) (Supplementary Table S4). The ferroptosis-related lncRNAs were visualized in a gene-lncRNA co-expression network (Figure 1D). As shown in the heatmap (Figure 1E) and volcano plot (Figure 1F), there were 161 FDELs (130 upregulated and 31 downregulated).

### Construction of the lncRNA signature

**Figure 2:**
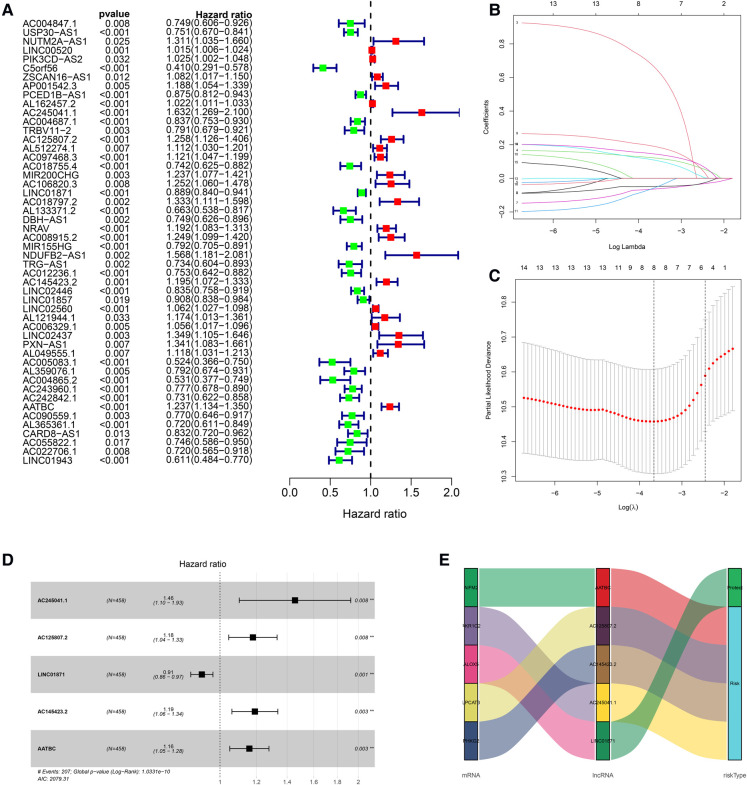
Construction of the prognostic ferroptosis-related lncRNA signature. (**A**) Univariate Cox regression revealed that 50 of the 161 FDELs were associated with OS. (**B**) LASSO regression identified 8 FDELs from the 50 lncRNAs, Dynamic LASSO coefficient profiling. (**C**) Selection of the tuning parameter (lambda); and the dashed lines on the left and right indicate the “lambda. min” and “lambda.1se” criteria, respectively. (**D**) Stepwise Cox regression finally identified 5 FDELs (AATBC, AC145423.2, LINC01871, AC125807.2, and AC245041.1) to construct the risk score (*P *< 0.001). (**E**) Sankey diagram shows the associations among FDELs, FDEGs, and risk types.

The univariate Cox regression analysis revealed that 50 of the 161 FDELs were significantly associated with OS (Figure 2A). LASSO regression revealed 8 of these 50 lncRNAs to be key to prognosis (Figures 2B,C). Stepwise multivariate Cox regression finally identified 5 lncRNAs (AATBC, AC145423.2, LINC01871, AC125807.2, and AC245041.1) for construction of the prognostic lncRNA signature (Figure 2D). Among these 5 lncRNAs, only LINC01871 was a protective factor, whereas the other 4 were risk factors (Figure 2E). The results of the univariate and multivariate Cox regression analyses of the 5 lncRNAs in the training cohort are shown in Supplementary Table S3. The risk score was calculated as follows: Risk score = 0.147 × expression of AATBC + 0.175 × expression of AC145423.2 - 0.091 × expression of LINC01871 + 0.165 × expression of AC125807.2 + 0.376 × expression of AC245041.1. All patients were divided into high- or low-risk group based on the median risk score of 0.995.

### Validation of the lncRNA signature

**Figure 3:**
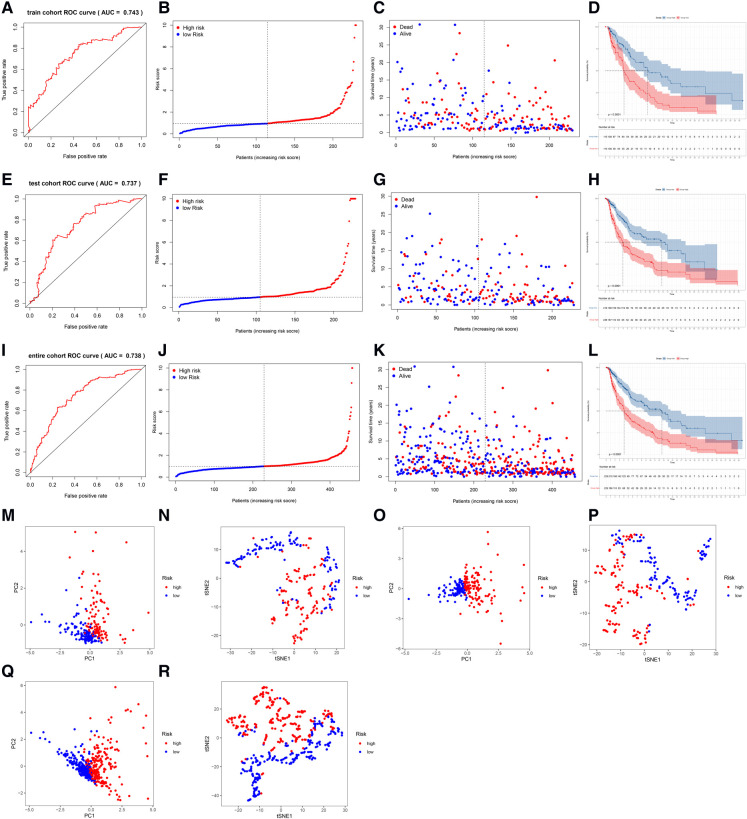
Evaluation and validation of the lncRNA signature in the training, testing, and entire cohorts. (**A**) AUC of the lncRNA signature for predicting 5-year survival in the training cohort. (**B**) The rank of the calculated risk score in the training cohort. (**C**) The survival status and survival time in the training cohort. (**D**) Kaplan-Meier survival curves of the high-risk patients and low-risk patients in the training cohort. (**E–H**) The lncRNA signature was validated in the testing cohort. (**I–L**) The lncRNA signature was validated in the entire cohort. (**M,N**) PCA and t-SNE analysis of the training cohort. (**O,P**) PCA and t-SNE analysis of the test cohort. (**Q,R**) PCA and t-SNE analysis of the entire cohort.

The AUC of the lncRNA signature for predicting 5-year OS in the TCGA training cohort was 0.743 (Figure 3A), indicating that the lncRNA signature offered accurate prediction. The mortality rate increased and survival time decreased as the risk score increased (Figures 3B,C). The OS rate of the high-risk group was significantly poorer than that of the low-risk group (Figure 3D). The ability of the 5-lncRNA signature to discriminate between high- and low-risk patients was validated in the testing cohort (Figures 3E–H) and entire TCGA cohort (Figures 3I–L). PCA and t-SNE analyses confirmed that the risk score had reliable clustering ability in the training cohort (Figures 3M,N), testing cohort (Figures 3O,P), and entire cohort (Figures 3Q,R).

### Independence predictors and comprehensive nomogram

**Figure 4:**
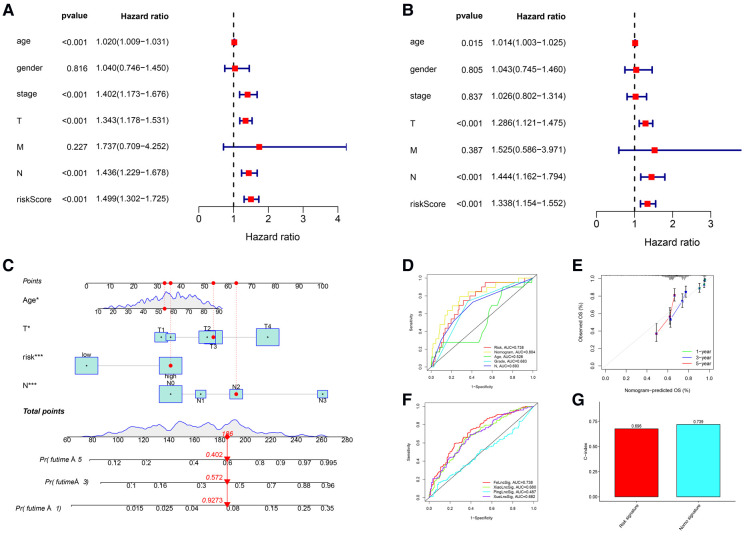
Independent prognostic value of the lncRNA signature and the comprehensive nomogram. (**A**) Univariate Cox regression identified individual factors related to OS. (**B**) Multivariate Cox regression identified independent predictors of OS. Our lncRNA signature was an independent predictor of OS with a hazard ratio of 1.338 (95% CI, 1.154-1.552, *P *< .001). (**C**) The nomogram including the lncRNA signature, age, T stage, and N stage for the practical prediction of the prognosis of CM patients. (**D**) AUCs of nomogram, signature and other clinicopathological characteristics for predicting 3-year OS. (**E**) A calibration curve analysis showed that the nomogram predictions were consistent with actual observations. (**F**) The ROC comparison of the studied signature with existing signatures. (**G**) The C-index of Nomogram.

The clinicopathological features of all 471 CM patients are shown in Supplementary Table S5. The HRs of the risk score in the univariate and multivariate Cox regression analyses were 1.499 (95% CI: 1.302–1.725, *P* < .001) and 1.338 (95% CI:1.154–1.552, *P* < .001), respectively (Figures 4A,B), indicating that the lncRNA signature was an independent predictor of OS.

We developed a nomogram that included the risk score and traditional clinicopathological factors for clinical use. The 1-, 3-, and 5-year survival probabilities for CM patients predicted with the nomogram were 92.7%, 57.2%, and 40.2%, respectively (Figure 4C). The AUC of the risk score for predicting 3-year OS (0.713) was greater than the AUCs of other clinicopathological features for predicting 3-year OS (684–0.693), indicating that the risk score had a better predictive ability (Figure 4D). In addition, the AUC of a prediction model that included the statistically significant variables in the multivariate analysis (i.e., risk score, age, T stage, and N stage) was 0.804, indicating that the novel risk score significantly increased the predictive accuracy of nomogram construction. The calibration curve analysis demonstrated excellent agreement between nomogram predictions and actual observations, indicating that the nomogram was stable and accurate in predicting the prognosis of CM (Figure 4E). Finally, the comparison of signature with other models showed that our AUC was superior to the existing signatures (Figure 4F). The calculation of C-index of Nomogram and signature were performed to reflect the validity of the nomogram and model, and two results are respectively 0.696 and 0.739 (Figure 4G).

### GSEA

**Figure 5:**
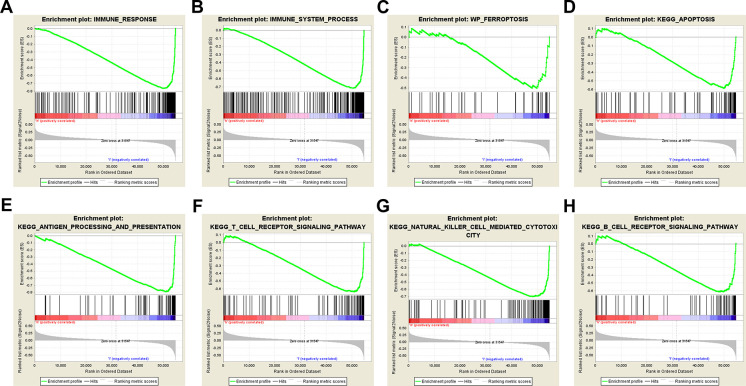
Gene set enrichment analysis. Immune-related and ferroptosis-related pathways were significantly enriched in the low-risk group. (**A**) Immune system process. (**B**) Immune response. (**C**) Ferroptosis. (**D**) Apoptosis. (**E**) Antigen processing and presentation. (**F**) T-cell receptor signaling pathway. (**G**) Natural killer cell-mediated cytotoxicity. (**H**) B-cell receptor signaling pathway.

The results of GSEA showed that ferroptosis, apoptosis, immune system process, immune response, antigen processing and presentation, T-cell receptor, B-cell receptor, and natural killer cell-mediated cytotoxicity pathways were inhibited in the high-risk group (Figures 5A–H). The lower level of ferroptosis and immune response in the high-risk group suggested that the association between ferroptosis and the immune TME play an important role in CM prognosis; therefore, we further compared immune cell infiltration and functions between the high- and low-risk groups.

### TME assessment

**Figure 6:**
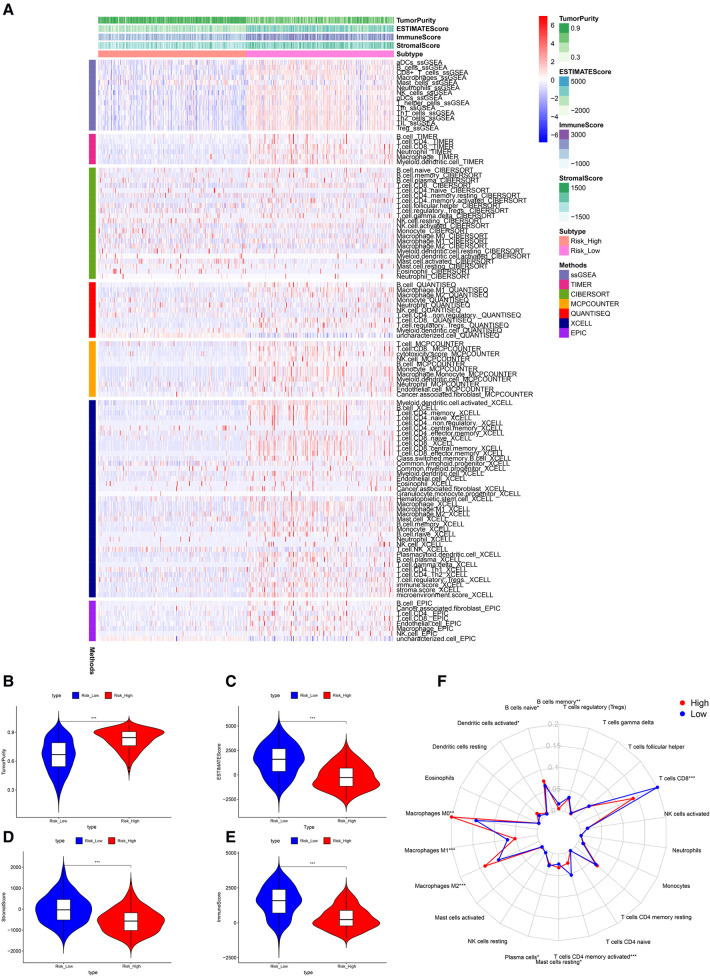
Tumor microenvironment analyses. (**A**) Immunity heatmap including the Tumor purity score, ESTIMATE score, immune score, stromal score and 7 other algorithms (ssGSEA, TIMER, CIBERSORT, MCPcounter, QUANTISEQ, XCELL, and EPIC). (**B**) The high-risk group had significantly higher tumor purity. (**C–E**) The high-risk group had significantly lower ESTIMATE, stromal, and immune scores. (**F**) A radar plot shows that the high-risk group had significantly fewer CD8+ T cells, CD4+ memory activated T cells and M1 macrophages, but more M0 and M2 macrophages. *P < 0.05; **P < 0.01; ***P < 0.001.

**Figure 7:**
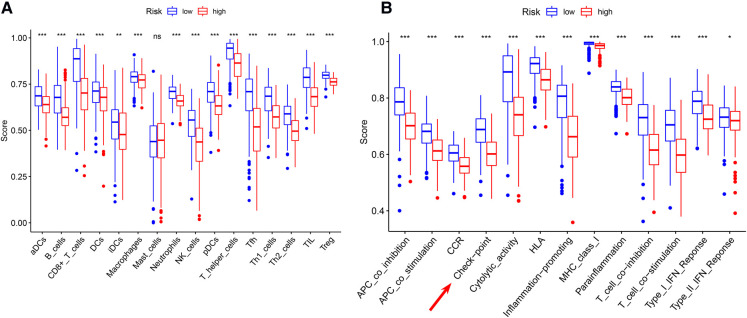
ssGSEA analyses (**A**) comparison of ssGSEA scores for 16 immune cells between the high- and low-risk groups. (**B**) A comparison of the 2 groups’ ssGSEA scores for 13 immune functions indicates that the high-risk group had a significantly lower the checkpoint score. *P < 0.05; **P < 0.01; ***P < 0.001.

Significant differences in immune cell infiltration between the high- and low-risk groups are illustrated in Figure 6A. Compared with the low-risk group, the high-risk group had significantly higher tumor purity (Figure 6B), but lower ESTIMATE scores (Figure 6C), stromal scores (Figure 6D), and immune score (Figure 6E), indicating that the high-risk group had a lower immune response. The high-risk group also had significantly fewer CD8+ T cells, CD4+ memory-activated T cells and M1 macrophages, but more M0 and M2 macrophages (Figure 6F). In addition, a comparison of ssGSEA scores for immune cells confirmed that the high-risk group had significantly fewer B cells, T cells, dendritic cells, macrophages, and nature killer cells than the low-risk group did (Figure 7A). ssGSEA score for immune functions demonstrated that pathways related to immune checkpoints, antigen-presenting cell, cell chemokine receptors, inflammation regulation, human leukocyte antigen, major histocompatibility complex, T cell functions, and type I/II interferon response were significantly downregulated in the high-risk group (Figure 7B).

### ICI efficacy

**Figure 8:**
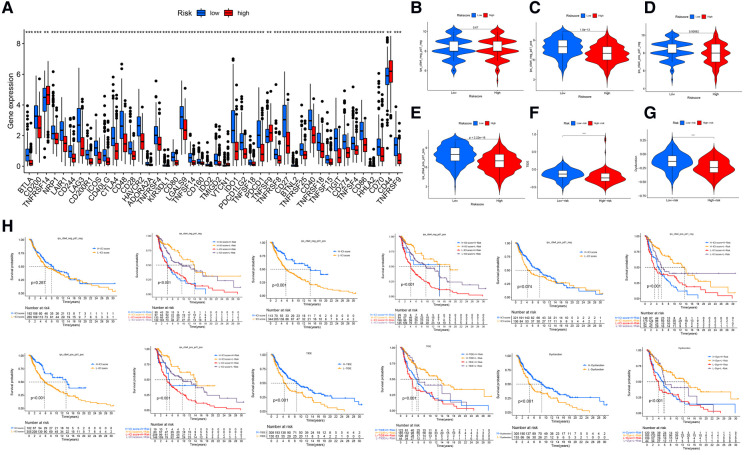
Immune checkpoint differences. (**A**) The expression of 44 immune checkpoint genes differed significantly between the high- and low-risk groups. The low-risk group had significantly higher expression of CD274 (*PDL-1*) and *CLAT-4* (*P* < .001). (**B**) The difference in ICI efficacy was not significant between the 2 groups if PD-1 and CLAT4 were both negatively expressed. (**C–E**) Low-risk patients with PD-1 or CTLA4 expression were likely to respond better to ICIs than high-risk patients were. (**F,G**) The TIDE and Dysfunction scores were higher in low-risk group than high-risk group. (**H**) The prognostic performance in immunotherapy cohorts.

The differences in the expression of 44 ICI-related genes between the high- and low-risk groups are shown in Figure 8A. The high-risk group had significantly lower expression levels of *PD-1* and *CTLA-4*. Among patients whose disease expressed either of these ICI-related genes, low-risk patients were likely to have a better response to ICIs than high-risk patients were, through the Cancer Immune Atlas (TCIA) database (Figures 8B–E). These results suggest that a higher level of ferroptosis facilitated a higher immune response in the TME, leading to increase the efficacy of ICIs in CM patients. Then, we also used TIDE (Tumor Immune Dysfunction and Exclusion) score to evaluate the response to immunotherapy, and the results showed TIDE and Dysfunction scores were higher in low-risk group than high-risk group (Figures 8F,G). The prognostic performance in immunotherapy cohorts were displayed through survival cures, indicating that cohorts with low risk combined with high ICI score have the better prognosis than other cohorts, especially with *PD-1* expression positive (Figure 8H).

### Tumor Mutational Burden (TMB) and drugs sensitivity analysis

**Figure 9:**
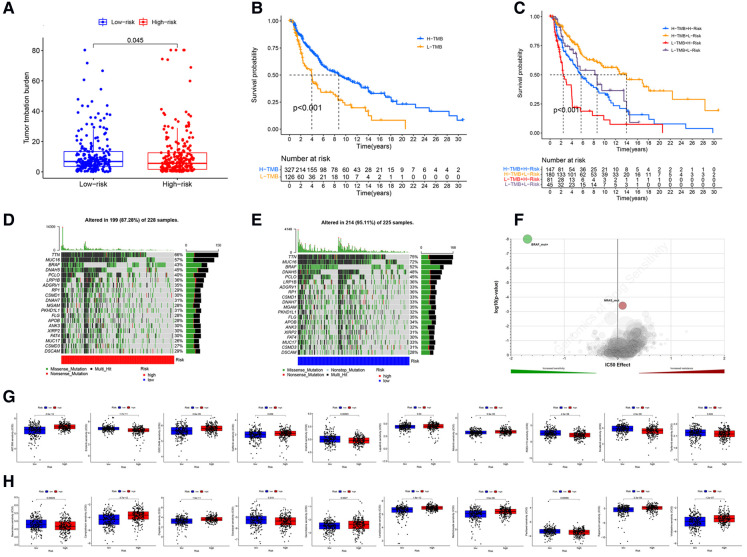
TMB and drugs sensitivity analysis. (**A**) The TMB in different risk groups. (*P* < .001). (**B,C**) The prognostic performance of TMB scores in survival cures. (**D–E**) The mutation rates of the top 20 genes in different risk groups. (**F**) The IC50 score with mutation. (**G**) The target drugs sensitivity in different risk groups. (**H**) The Chemotherapy drugs sensitivity in different risk groups.

The analysis demonstrated that the TMB had significantly difference between low- and high- risk groups (Figure 9A), and TMB has a significant impact on patient prognosis between low- and high- risk groups (Figures 9B,C). Then, we then identified the top 20 genes with the highest mutation rates in the two risk subgroups. The mutation rates of TTN, MUC16, BRAF, and DNAH5 were higher than 30% in both groups (Figures 9D,E). Mutation of the TTN gene was more common in the high-risk subgroup. Genetic mutations can affect the tumor response to chemotherapy and targeted therapy (Figure 9F); therefore, we investigated the association between the risk model and the efficacy of chemotherapy and targeted therapy drugs in patients with CM. We listed 10 common target drugs (Figure 9G) and 10 Chemotherapy drugs (Figure 9H) used for CM, such as sorafenib, Imatinib, ABT.888 (Veliparib), Docetaxel, and Paclitaxel (Figure 9H). Significant differences in the estimated IC50 between the two risk groups were observed, which suggest that the risk model might be used to identify potential biomarkers for chemotherapy and targeted therapy sensitivity.

## Discussion

We performed comprehensive analyses utilizing the TCGA, GTEx, and FerrDb databases and identified 5 key lncRNAs to construct a risk score that stratified CM patients into high- and low-risk groups. The lncRNA signature was an independent prognostic predictor of OS with fair accuracy. The nomogram that included the lncRNA signature and other clinicopathological factors had good accuracy and reliability in predicting the OS of CM patients. The high-risk group was associated with lower ferroptosis, immune response, immune cell infiltration and immune function. High-risk patients had also lower expression of ICI-related genes such as *PD-1* and *CTLA-4*, and thus had poorer responses to ICIs. These results collectively demonstrate that this novel ferroptosis-related lncRNA signature can predict the prognosis of CM and the efficacy of ICIs in CM patients.

Ferroptosis-related lncRNA signatures have considerable predictive value for the prognosis of CM. Recent studies have developed several lncRNA signatures related to autophagy (40) and immunity (41–44) to predict the prognosis of CM. In these studies, the numbers of lncRNAs used for signature construction ranged from 3 to 24 (Supplementary Table S6). Similarly, we constructed a risk score based on 5 lncRNAs we identified (AATBC, AC145423.2, AC125807.2, AC245041.1, and LINC01871). The regulatory roles and molecular mechanisms of these 5 lncRNAs have been shown in previous studies.

AATBC (apoptosis-associated transcript in bladder cancer) was originally discovered in bladder cancer by Zhao et al. (45), who found that AATBC overexpression of AATBC is positively correlated with tumor grade and stage. A transcriptomic analysis of high-throughput sequencing data (46) confirmed that AATBC can harbor bladder cancer-related miRNA recognition elements. Wang et al. (47) identified a multi-RNA-type-based signature that included AATBC for predicting recurrence-free survival in patients with uterine corpus endometrial carcinoma. Tang et al. (48) found that AATBC overexpression promotes the migration and invasion of nasopharyngeal carcinoma cells *in vitro*, as well as their metastasis *in vivo*, through the miR-1237-3p-PNN-ZEB1 axis, leading to poor survival. Tang et al. (48) showed that AATBC is highly expressed in breast cancer and promotes cancer migration and invasion by activating the YAP1/Hippo signaling pathway through the AATBC-YBX1-MST1 axis. Zhang et al. (49) demonstrated that AATBC overexpression promotes the proliferation and migration of prostate cancer cells *via* the miR-1245b-5p-CASK axis. The role of AATBC in CM was reported by Yan et al. (50), who constructed a prognostic model with 7 gene instability–related lncRNAs, including AATBC. Their cell experiments verified that AATBC knockdown can inhibit the proliferation and invasion of CM. In accordance with these studies, we found that AATBC is a risk factor for poor prognosis in CM patients.

Unlike that of AATBC, the regulatory roles of the other 3 risk factors (AC145423.2, AC125807.2 and AC245041.1), have been rarely reported. Xuan et al. (51) identified 7 autophagy-related lncRNAs including AC145423.2 to build a risk score, for dividing clear cell renal cell carcinoma patients into low- and high-risk groups. Hou et al. (52) constructed a competing endogenous RNA network that included AC125807.2 and 11 other lncRNAs, and found AC125807.2 to be an independent prognostic predictor of OS in patients with lung adenocarcinoma. Huang et al. (53), who established a ferroptosis-related lncRNA signature, which included AC245041.1, for gastric cancer, found that higher AC245041.1 expression is correlated with poorer OS. In agreement with these studies, our results showed that AC145423.2, AC125807.2 and AC245041.1 are associated with poor prognosis in CM patients. However, the mechanism underlying how lncRNA regulates ferroptosis remains elusive and requires further investigation.

Previous studies have shown that LINC01871 is related to stemness, autophagy, and immunity in breast, gastric, cervical, and endometrial cancers. For example, Li et al. (54) identified 12 stemness-related lncRNA including LINC01871, to predict the prognosis of breast cancer. In a second, they identified 11 autophagy-related lncRNAs including LINC01871, in breast cancer (55). Ma et al. (56) established a signature comprising 8 immune-related lncRNA including LINC01871, to predict the survival of breast cancer patients. Moreover, LINC01871 has a strong positive correlation with ICI-related genes such as *CTLA4* and *PD1*, which suggested that the lncRNAs has critical roles in the immune response and immunotherapy outcomes. Wu et al. (57) identified 5 autophagy-related lncRNAs including LINC01871, to construct a risk score and a nomogram to predict the prognosis of breast cancer patients. Mathias et al. (58) investigated the role of LINC01871 in 5 molecular subtypes of breast cancer and found that LINC01871 is associated with immune response activation and favorable OS in patients with basal-like breast cancers. He et al. (59), in a multi-omics data analysis, identified molecular features (e.g., genes, miRNAs, lncRNAs, proteins, pathways) that correlated with tumor immunity in gastric cancer. They found that LINC01871 was positively associated with cancer immunity (expression levels of CD8+ T cell, cytolytic immune activity, and PD-L1), which suggests that LINC01871 is a useful biomarker for assessing immunotherapy response. In addition, Chen et al. (60) constructed a signature of 6 immune-related lncRNAs, including LINC01871, to predict the prognosis of cervical cancer. Similarly, Wang et al. (61) identified a signature of 5 autophagy-related lncRNAs, including LINC01871, to predict the prognosis of endometrial cancer patients. Together, these studies demonstrate that LINC01871 is positively associated with the immune response, and thus a protective factor for cancer prognosis. This is consistent with our finding that LINC01871 is the only ferraptosis-related protective factor for OS in CM.

The AUCs of lncRNA signature for OS prediction significantly varied in previous studies, and (Supplementary Table S5). Our ferroptosis-related lncRNA signature had AUCs for predicting 5-year OS of 0.743, 0.737, and 0.738 in the training, testing, and entire cohorts, respectively. These results are in line with those of the studies by Yoshihara et al. (17), Xiao et al. (62), and Wang et al. (42), whose signatures for predicting survival also had an AUCs of 0.7–0.8. And the HRs of lncRNA signatures ranged from 1.024 to 2.043 (*P* < 0.05 for all), also listing in Supplementary Table S5. In accordance with these studies' findings, our multivariate Cox regression analysis showed that our lncRNA signature had an HR of 1.338, indicating that the mortality risk of a CM patient would increase by 33.8% as the risk score increased by 1 point.

Our nomogram integrated the independent risk factors identified in the multivariate Cox regression analysis. This combination significantly increased the predictive accuracy of Ferroptosis-related signature in clinical application, as it had an AUC of 0.804 for predicting 3-year OS in the entire cohort. The calibration curve analysis demonstrated excellent agreement between nomogram predictions and actual observations, indicating that our nomogram was stable and accurate in predicting the prognosis of CM. Few others have developed nomograms for the prediction of CM prognosis. Although Xiao et al. (62) constructed a nomogram that included the same predictors as ours, they did not report the AUC of the nomogram or evaluate its performance. Tian et al. (63) noted that the AUCs of their nomogram for predicting 3-year OS were 0.816 and 0.740 in the training and testing cohorts, respectively, but their nomogram had more variables than ours and was not verified by calibration curve analysis.

The imbalance of immunity in the TME plays an important role in the development and progression of CM. Ferroptosis is associated with the immune TME and the response of CM to immunotherapies (64). Specific ferroptosis-related lncRNAs can regulate the TME by affecting antigen release/presentation, immune activation, immune cells migration/infiltration, and even enhancing the efficacy of ICIs (65–68). Previous studies showed that patients with lower immune cell (e.g., CD8 + T cells, M1 macrophages) infiltration and function have poorer survival (69). For example, Wang et al. (42) found that high-risk CM patients have lower immune functions than low-risk patients do. Xiao et al. (62) showed that high-risk CM patients have lower levels of CD8+ T cells, CD4+ memory-activated T cells, and M1 macrophages, but higher levels of M0 macrophages. Their results were consistent with those of Ping et al. (41), who found that high-risk CM patients have lower levels of B cells, CD4+ T cells, monocytes, and CD8+ T cells, but higher levels of M0 macrophages. Thus, it may have lower responses to chemotherapeutics (i.e., cisplatin, vinblastine, paclitaxel) and ICIs. In accordance with those studies, the present study demonstrated that high-risk patients have significantly lower immune cell (e.g., CD8+ T cells) infiltration and immune function (e.g., immune checkpoint activation), possibly because immunotherapy-activated CD8+ T cells enhance lipid peroxidation and ferroptosis as a cytotoxic mechanism in CM cells and ultimately promote the efficacy of anti-CTLA-4 and anti-PD-1 therapies (70).

The present study had several limitations. First, the 5 lncRNAs were identified and validated using a single data source (TCGA). It would have been better if we tested the prognostic value of these lncRNAs in another independent patient cohort. its prognostic values were tested in another independent patient cohort. Second, the molecular mechanism of these lncRNAs remains unclear and requires further validation using cellular/ molecular biological experiments. Despite these limitations, this is the first study to identify a ferroptosis-related lncRNA signature for the prediction of prognosis and immunotherapy efficacy in CM patients. Owing to its relevance to the immune TME and ICI-related gene expression, these ferroptosis-related lncRNAs may be potential biomarkers and therapeutic targets for CM. The lncRNA signature may help improve the efficacy of personalized immunotherapy.

Our novel ferroptosis-related lncRNA signature was able to accurately predict the prognosis of CM patients and their outcomes. The novel ferroptosis-related lncRNA signature was able to accurately predict the prognosis and outcome of ICI therapies. These ferroptosis-related lncRNAs might be potential biomarkers and therapeutic targets for CM.

## Data Availability

The original contributions presented in the study are included in the article/**Suplementary Material**, further inquiries can be directed to the corresponding author/s.
