# Supplementary material for: Ferroptosis-related lncRNA signature predicts prognosis and immunotherapy efficacy in cutaneous melanoma
Source: Front Surg. 2022 Jul 21;9:860806. doi: 10.3389/fsurg.2022.860806 (PMC9354448; doi:10.3389/fsurg.2022.860806)
Supplement: Supplementary file 1 [file Data_Sheet2_v1.pdf]

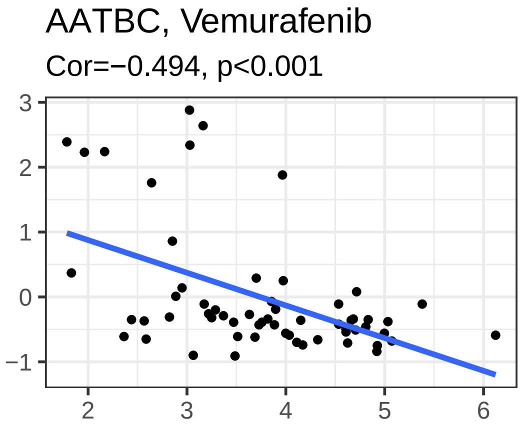
AATBC, Vemurafenib
Cor=−0.494, p<0.001
3
2
1
0
−1
2
3
4
5
6

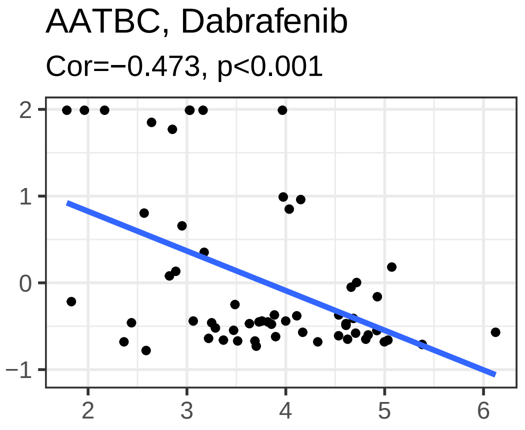
AATBC, Dabrafenib
Cor=−0.473, p<0.001
2
1
0
−1
2
3
4
5
6

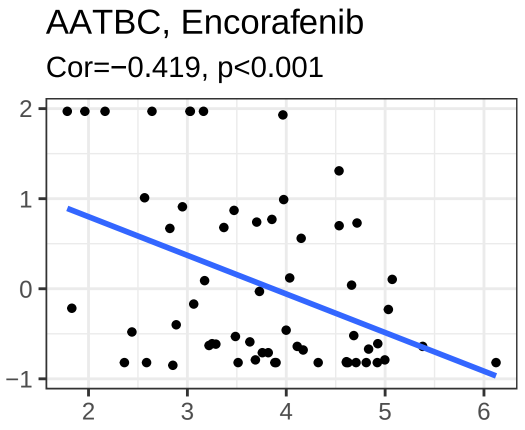
AATBC, Encorafenib
Cor=−0.419, p<0.001
2
1
0
−1
2
3
4
5
6

AATBC, Selumetinib

Cor=−0.399, p=0.002

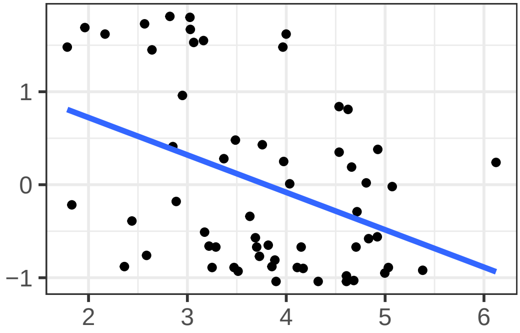

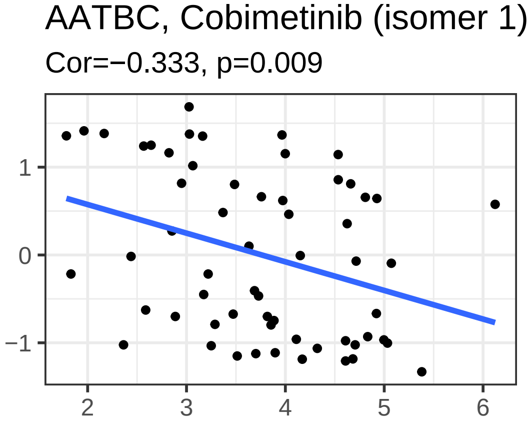
AATBC, Cobimetinib (isomer 1)
Cor=−0.333, p=0.009
1
0
−1
2
3
4
5
6

AATBC, Imatinib

Cor=0.329, p=0.010

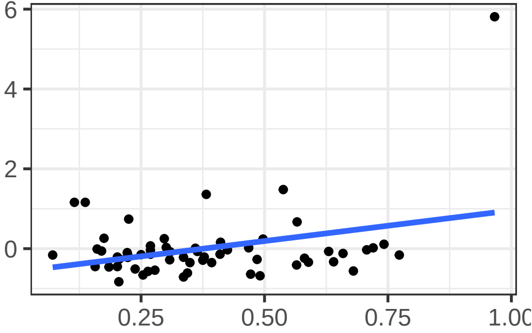

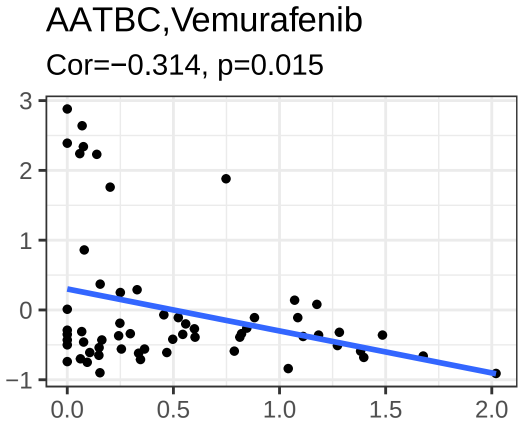

AATBC,Vemurafenib
Cor=−0.314, p=0.015
3
2
1
0
−1
0.0
0.5
1.0
1.5
2.0

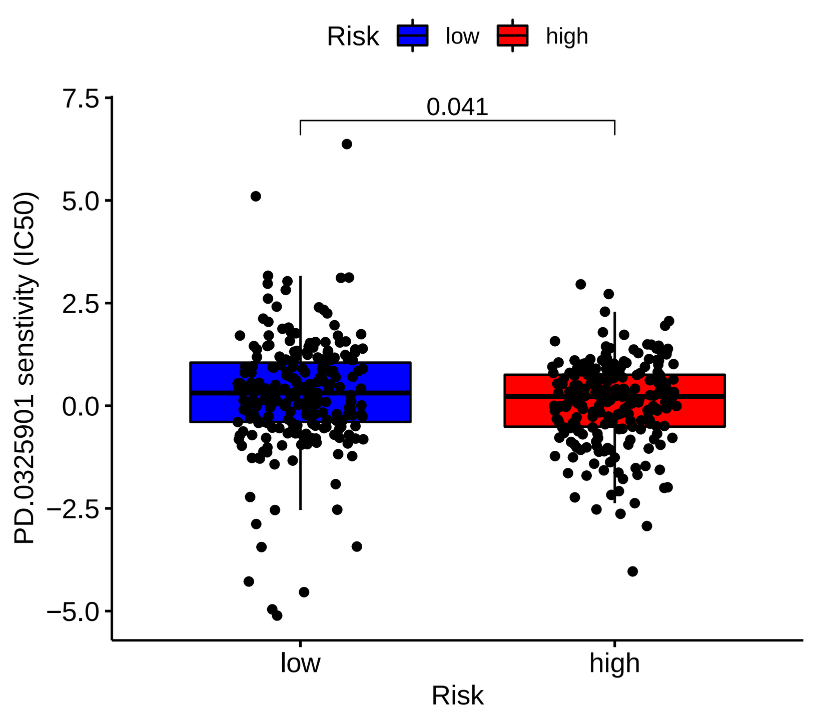

Risk
low
high
0.041
PD.0325901 senstivity (IC50)
7.5
5.0
2.5
0.0
−2.5
−5.0
low
high
Risk

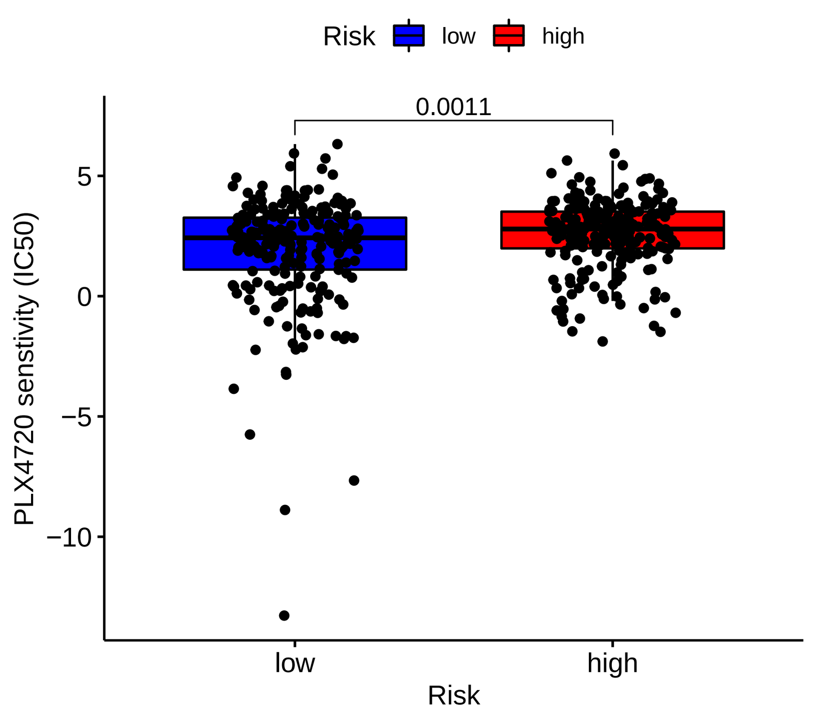

Risk
low
high
0.0011
5
0
−5
−10
PLX4720 senstivity (IC50)
low
high
Risk

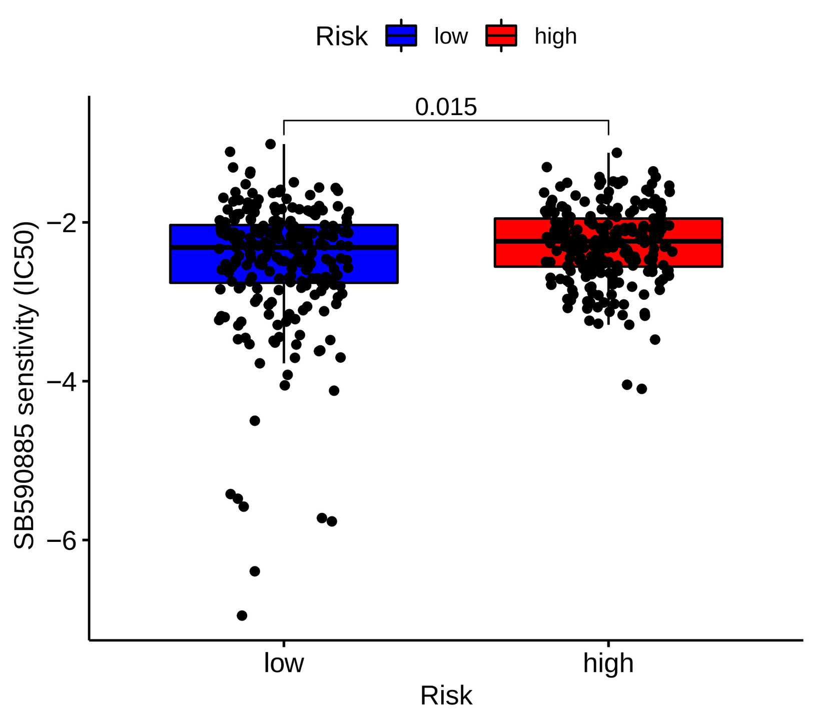

Risk
low
high
0.015
−2
−4
−6
SB590885 senstivity (IC50)
low
high
Risk

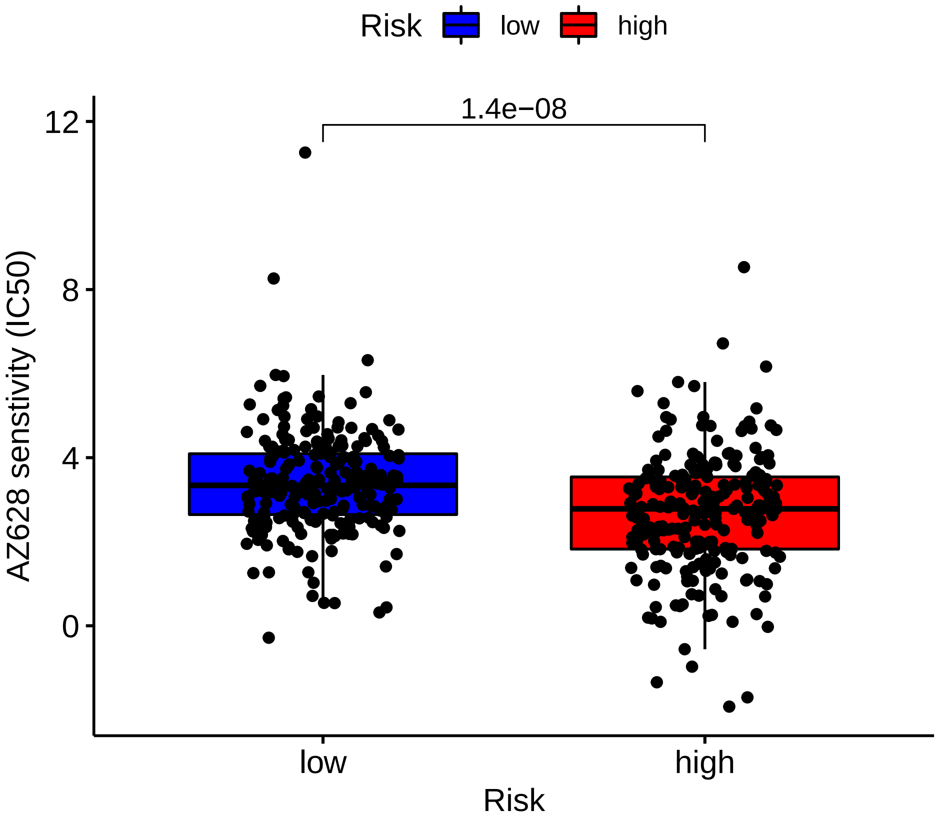
Risk
low
high
1.4e−08
AZ628 senstivity (IC50)
12
8
4
0
low
high
Risk

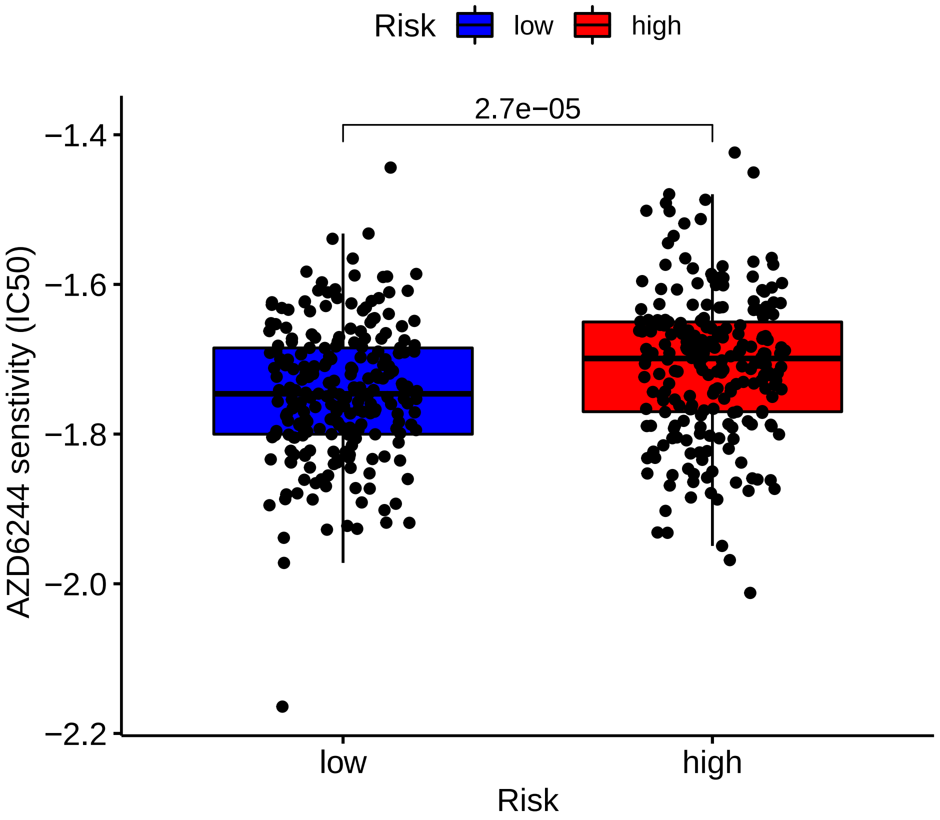

Risk
low
high
2.7e−05
AZD6244 senstivity (IC50)
−1.4
−1.6
−1.8
−2.0
−2.2
low
high
Risk

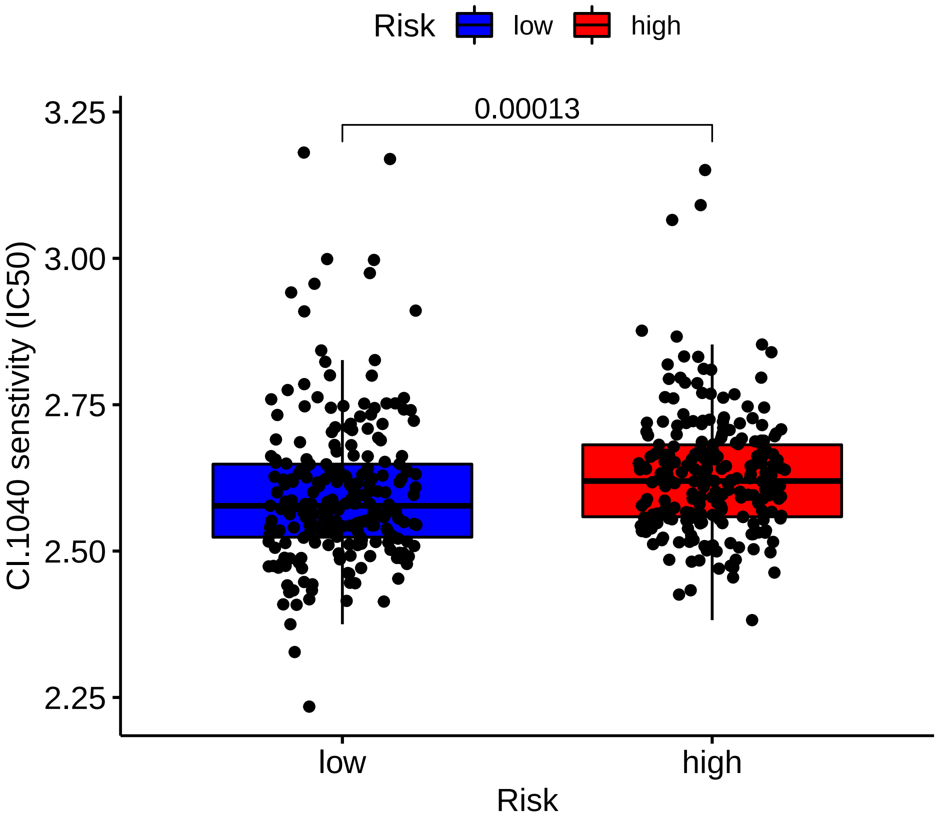
Risk
low
high
0.00013
CI.1040 senstivity (IC50)
3.25
3.00
2.75
2.50
2.25
low
high
Risk
